# Preventing Gender-Based Homelessness in Canada During the COVID-19 Pandemic and Beyond: The Need to Account for Violence Against Women

**DOI:** 10.1177/10778012211034202

**Published:** 2021-09-17

**Authors:** Alexa R. Yakubovich, Krys Maki

**Affiliations:** 1 University of Toronto - Dalla Lana School of Public Health, Toronto, Canada; 2 Women's Shelters Canada, Ottawa, Canada

**Keywords:** intimate partner violence, housing, homelessness, women, Canada

## Abstract

The coronavirus disease of 2019 (COVID-19) pandemic has led to increases in intimate partner violence (IPV), a leading cause of women's homelessness. Although the Canadian Government provided emergency funding to the violence against women and housing and homelessness sectors in response to COVID-19, Canada lacks a national legislative and funding framework to support coordinated prevention efforts. We review the context of IPV and homelessness among women and international policy exemplars. We then propose several starting points for developing a Canadian strategic framework, including adopting inclusive definitions of IPV and homelessness as well as evaluating a broad continuum of IPV-housing options and intersectoral partnership models.

The intersections of intimate partner violence (IPV), homelessness, and the coronavirus disease of 2019 (COVID-19) pandemic necessitate a coordinated public health response strategy. As in prior infectious outbreaks, the COVID-19 pandemic has been associated with higher risks of violence against women, including IPV (Wenham et al., 2020). In addition to its severe health consequences, IPV is a leading cause of women's homelessness—which both precipitates and exacerbates poor health conditions (Daoud et al., 2016; Schwan et al., 2020). Although there are successful local examples, Canada's national response efforts have been largely siloed, limited by the nation's lack of policy frameworks on preventing gender-based violence and missing and murdered Indigenous women and girls. These gaps must be urgently addressed through a coordinated systems approach.

In this activist/advocate note, we outline the context of gender-based violence and homelessness in Canada prior to COVID-19 and how the pandemic has exacerbated structural vulnerabilities for women. We propose that Canada needs a national legislative and funding framework that supports intersectoral coordination to maximize prevention efforts for women's homelessness and IPV during and beyond the pandemic. We discuss several starting points for building this framework based on successful local examples and international approaches, including adopting inclusive definitions of IPV and homelessness and evaluating a broad continuum of structural solutions. The COVID-19 pandemic has highlighted the gaps in public health policy for marginalized communities and presents a critical opportunity to restructure for a more equitable future of public health. Doing so requires countries to not only reflect inwards, but to consider successful policy and practice internationally, including areas of implementation that can be further improved.

## What was the State of IPV and Gender-Based Homelessness Before the COVID-19 Pandemic?

Although people of all genders experience IPV, women and gender-diverse populations tend to experience more severe IPV and more physical, psychological, and socioeconomic impacts (Burczycka et al., 2019; Canadian Centre for Justice Statistics, 2016; Stark, 2007). IPV is a major determinant of women's homelessness, which frequently remains “hidden” compared to men's homelessness (Bernas et al., 2019; Maki, 2017; Schwan et al., 2020; Sev’er, 2002; Tutty et al., 2013). Women at risk of homelessness often employ survival tactics not captured in routine data collection on homelessness (e.g., relying on provisional or overcrowded accommodation, staying with violent partners, exchanging sex for shelter). Violence against women shelters, which are chronically underfunded and often running at maximum capacity, are also typically excluded from these counts. Living or staying in homeless shelters (largely occupied by men and similarly facing capacity and funding shortages) and on the streets can increase women's risk of experiencing violence, state surveillance, criminalization, and child apprehension, particularly for Indigenous and racialized women (Schwan et al., 2020). As a result, IPV survivors are often not counted in definitions of homelessness—especially “chronic homelessness,” the target of many homelessness policies (ESDC, 2020).

Most specialized housing supports available to women experiencing IPV in Canada are mandated for short-term stays (i.e., <2 months) (Maki, 2020). Few options exist for longer-term housing, especially those with wraparound supports, critical for IPV survivors (Baker et al., 2010; Botein & Hetling, 2016; Reid et al., 2020; Sullivan, 2018), including transitional or second-stage shelters (allowing 1- to 2-year stays) and permanent supportive housing solutions. A Canadian National Housing Strategy was formed in 2017, with long-term housing supports focused on Housing First programming. Housing First prioritizes rapid rehousing and in practice has traditionally been targeted towards those experiencing standard definitions of chronic homelessness, largely without addressing women's unique long-term housing needs (Schwan et al., 2020). Reflecting this focus, evaluations of Housing First in Canada, while showing positive mental health and housing outcomes, have tended to rely on male-majority samples and a gender-neutral framework (Oudshoorn et al., 2018). These service and research gaps are exacerbated by the lack of a National Action Plan on gender-based violence, long advocated to increase investment in prevention and response strategies to IPV and other forms of violence against women and gender-diverse people (e.g., Women’s Shelters Canada, 2019).

## How Have Burdens of Violence, Homelessness, and COVID-19 Intersected Among Women?

Emerging evidence from the COVID-19 pandemic indicates that IPV has increased over the last year due to stressors like income loss or precarious employment, service disruptions, and lockdown measures (Bourgault et al., 2021; Piquero et al., 2021). In Canada, national surveys of violence against women service providers have demonstrated significant pandemic-related impacts, including challenges in delivering services (e.g., demand for personal protective equipment, physical distancing measures, technological support) and women accessing them (e.g., who are sheltering at home with violent partners) (Trudell & Whitmore, 2020; Women’s Shelters Canada, 2020). A significant proportion of respondents in these surveys (34%–52%) observed increases in the severity of violence experienced by survivors and mental health challenges (e.g., increased suicidal ideation).

Changes in the risk and severity of IPV are exacerbated by a collision of structural vulnerabilities disproportionately impacting women during this pandemic, as in prior public health emergencies (Wenham et al., 2020). These gender-based impacts include: health (e.g., women comprise the majority of at-risk frontline and service sectors); unpaid work (e.g., childcare); and economic impacts (e.g., due to women occupying the majority of precarious and low-income employment) (United Nations, 2020). In Canada, Indigenous women face even greater risks of contracting coronavirus and experiencing IPV, homelessness, and other health and socioeconomic impacts (Palmater, 2020). These structural inequities—rooted in the inequitable distribution of power and resources in society—are the well-established “causes of causes” of poor health and wellbeing and must be ameliorated through structural change (i.e., policy and systems-level interventions) during COVID-19 and beyond (Link & Phelan, 1995).

## How Have Coordinated Response Strategies to IPV and Gender-Based Homelessness Been Applied Locally in Canada and in Other Countries?

Despite the gender-based and intersectional impacts of the COVID-19 pandemic, Canada paused development of its National Action Plans on gender-based violence and Missing and Murdered Indigenous Women and Girls as well as the National Indigenous Housing Strategy during the first 6 months of the pandemic. The Federal Government committed financial support in response to women's experiences of homelessness and violence in May 2020: $76 million for violence against women shelters (including off-reserve Indigenous shelters), sexual assault centers, and nonprofit gender-based violence organizations; and $10 million for shelters supporting Indigenous women and children fleeing violence on reserves (Status of Women Canada, 2020). The housing and homelessness sector received $394 million during the pandemic for the Reaching Home program (the funding program for the National Housing Strategy) and $1 billion for a new Rapid Housing Initiative (Canada Mortgate and Housing Corporation, 2020). This emergency funding was critical (and more was committed in late 2020). Although the segmented funding is demonstrative of the historically distinct service systems of these two sectors (Baker et al., 2010; Botein & Hetling, 2016), the continued, overlapping challenges faced by these and other sectors during COVID-19 (and beyond) raise long-advocated for opportunities to work together for more sustainable and equitable impact. This requires permanent, coordinated investment and policy from all levels of government.

As the Canadian Government recommits to developing its National Action Plan on gender-based violence (with expert engagement, e.g., Dale et al., 2021), there are promising international examples of intersectoral collaboration that can inform its development and coordination with the National Housing Strategy. Major IPV and homelessness policy reforms, legislation, and collaborative efforts have driven the widening of housing supports available to IPV survivors internationally (Spinney & Blandy, 2011), including during the pandemic. For instance, in July 2020, the U.K. House of Commons passed the Domestic Abuse Bill after several years of development. Now signed into law, the Act includes several intersectoral measures relevant to IPV and homelessness, such as priority need for housing for all people experiencing IPV, flexible domestic abuse protection orders (allowing for longer-term prohibitions on contact or home occupation, and civil or criminal sanctions), and upholding lifetime tenancies when rehousing or granting new sole tenancies to social housing residents who are experiencing domestic abuse (UK Parliament, 2021). The Act further establishes the country's first statutory definition of domestic abuse, which includes not only physical or sexual violence but also emotionally abusive, financially abusive, coercive, or controlling behaviors, in line with international standards. Access to IPV-related services and supports across sectors is expected to be available to all those who meet this more inclusive definition, although a major area of contention among advocates has been the exclusion of migrant women from the Act's provisions (Women’s Aid, 2021).

The effectiveness of these new measures is as yet untested and they will require cross-sectoral training and education on understanding, identifying, and responding to a more inclusive typology of IPV, which are currently nonstatutory commitments of the Domestic Abuse Act (UK Parliament, 2021). This is a significant implementation challenge, especially given prior evaluations of IPV risk assessments, which have shown that practitioners without specialist training tend to underestimate nonphysical or sexual forms of abuse (Robinson et al., 2018). However, these advances will be facilitated by a national policy and practice context already operating with an intersectoral approach to IPV. This includes coordinated homelessness and IPV prevention legislation, such as allowing IPV survivors to be added to or granted new sole tenancies and the removal of violent partners in certain situations (Ministry of Housing Communities & Local Government, 2018; Spinney, 2012). Scotland has taken coordinated legislation even further in an amendment to its own Domestic Abuse Act, which became law in May 2021 (Scottish Parliament, 2021). The amendment, among other things, allows for a sole social tenancy in the partner's name to be transferred to the survivor's name. The U.K. practice context also includes ∼300 Multi-Agency Risk Assessment Conferences (MARACs) across the country, which entail monthly, coordinated case management of high-risk cases involving actors from multiple sectors, such as shelter, criminal justice, housing, and health care as well as independent domestic violence advocates (IDVAs) (Robbins et al., 2014). Notably, embedding IDVAs in health systems has proven to be a promising innovation for supporting the identification and referral of patients experiencing IPV, developed in the United Kingdom (Dheensa et al., 2020; Feder et al., 2011; Sohal et al., 2020), yet the lack of investment in the health care response to IPV has been a major criticism of the Domestic Abuse Act (Inter-Collegiate and Agency Domestic Violence Abuse, 2020). Finally, there are exemplar models of national intersectoral collaboration, such as the Domestic Abuse Housing Alliance, a partnership between domestic abuse services and housing providers that advocates for a suite of IPV housing options and accredits housing providers with IPV specialist training (Whole Housing Domestic Abuse, 2020).

The United Kingdom serves as one, albeit imperfect, example of how national and local systems innovations have strengthened intersectoral action for gender-based violence and homelessness. Others include Australia, which over the last decade has implemented transformative gender-based violence legislation at both the state and national levels (thus offering a parallel context to Canada), as well as New Zealand, which likewise implemented national legislation and is in the process of designing its National Action Plan to prevent family and sexual violence (Council of Australian Governments, 2019; Ministry of Justice, 2020; Spinney, 2012). As a result, there are a number of (particularly, long-term) supportive housing options available to those experiencing IPV in these and other countries, which remain widely unavailable in Canada (see Table 1). These examples are meant to demonstrate, nonexhaustively, the innovation that has occurred along the continuum of housing options available for IPV survivors, especially through collaboration across the violence against women and housing and homelessness sectors (e.g., see Baker et al., 2010; Botein & Hetling, 2016; Klein et al., 2021; Maki, 2019; Spinney, 2012; Whole Housing Domestic Abuse, 2020).

**Table 1.** Example Intervention Models in the Continuum of IPV-Housing Options by Availability in Canada Versus Internationally.

IPV-housing option	Widely available in Canada?^a^	If not, international exemplars
Emergency violence against women shelters	Yes	—
Violence against women transitional houses/second-stage shelters	Yes	—
Priority social housing/portable housing benefits, rental subsidies, and housing vouchers for women experiencing IPV	Yes	—
Third-stage shelters for IPV survivors, with longer temporary housing and programmatic support following second-stage shelter	No^b^	—
Domestic Violence Housing First or permanent supportive housing models that provide rapid rehousing with tailored services and advocacy for IPV survivors	No^c^	The United States of America, the United Kingdom
Stay at home models that support women to stay in their homes and the removal of violent partners, with home security upgrades and IPV support services	No	The United Kingdom, Australia, New Zealand
Flexible funding that provides short-term financial and advocacy support to IPV survivors to address barriers to securing housing stability	No	The United States of America, the United Kingdom
Reciprocal schemes that involve partnerships between social housing providers and specialist IPV services to rapidly relocate and rehouse tenants experiencing IPV	No	The United Kingdom

*Note*. These examples are meant to (nonexhaustively) demonstrate the innovation that has occurred across the continuum of housing options available for IPV survivors, especially through collaboration across the violence against women and housing and homelessness sectors (e.g., see, Baker et al., 2010; Botein & Hetling, 2016; Klein et al., 2021; Maki, 2019; Spinney, 2012; Whole Housing Domestic Abuse, 2020). A continuum of options is needed to meet the diversity of women's needs: no one option will be the best fit for all women. Further evaluation of what works best, how, and for whom is needed. The availability of safe and accessible public and private (rented and owned) housing is also critical and providers/landlords (where relevant) may benefit from IPV specialist training. IPV = intimate partner violence.

a “No” indicates that, while there are some local examples of these intervention models being evaluated or implemented in Canada, these have not been evaluated or implemented across the country.

b Limited availability in select provinces (British Columbia, Alberta, and Nova Scotia).

c Limited availability in select provinces (British Columbia and Ontario).

Despite a lack of large-scale investment in coordinated responses to IPV-related homelessness in Canada, locally practitioners have successfully committed to delivering longer-term options. For instance, a small-scale evaluation of Housing First adapted for women's homelessness needs in the province of Ontario found that six of 10 participants who had experienced complex histories of homelessness, violence, and trauma were housed at the end of the 2-year funding period, although the evaluators highlighted the need for a stronger integration with community services (Oudshoorn et al., 2018). Evaluations of gender or IPV-specific long-term supportive housing solutions remain limited. A recent scoping review of women's housing needs in Canada did not identify evaluations of long-term housing interventions for women (in general) or IPV survivors (in particular) that were sustained beyond the evaluation period (Schwan et al., 2020). A systematic review of U.S.-based evaluations likewise found limited evidence (Klein et al., 2021). This raises important questions of what will work best, how, and for whom, highlighting the need for the expansion of policy and programming to be coupled with strong monitoring and evaluation frameworks, co-designed with the input of IPV experts, as discussed below.

## How Can Coordinated Intersectoral Action be Systematically Implemented in Canada During the COVID-19 Pandemic and Beyond?

To advance a coordinated intersectoral approach to preventing gender-based homelessness and violence in Canada, inclusive and standardized definitions of IPV and homelessness should be applied across (and within) jurisdictions, including in national funding streams. Legal definitions of IPV currently vary across Canada, often barring survivors from critical supportive services (e.g., justice, social protection) if they have not experienced specific definitions of physical or sexual violence (Koshan et al., 2020; Watson Hamilton, 2019). Regarding homelessness, the Canadian Observatory on Homelessness proposed a national definition inclusive of those who are unsheltered, emergency sheltered, provisionally accommodated, and at risk of homelessness (including women experiencing IPV) (Canadian Observatory on Homelessness, 2017). Many aspects of this definition were adopted in the funding program underlying Canada's National Strategy (Reaching Home). However, only in response to COVID-19 have explicit statements been added of eligibility for violence against women shelters that encourage coordination between IPV and housing service providers in pandemic-response strategies (ESDC, 2020). The permanence of the inclusion of violence against women shelters in this funding eligibility is unclear and criteria of chronic homelessness—which exclude women who have not stayed in shelters for more than 6 months—problematically remain.

Moving forward, policies and programs related to homelessness should explicitly adopt an intersectional gender-based definition of homelessness that understands women's unique experiences, based on multiple and intersecting social locations: gender, sexual identity, race/ethnicity, Indigeneity, class, disability, and age (Bernas et al., 2019; Maki, 2017; Schwan et al., 2020; Sev’er, 2002; Tutty et al., 2013). This must include recognition of IPV survivors’ experiences of homelessness, including violence against women shelters, couch surfing, and living with abusive partners, during and beyond COVID-19. Likewise, the National Action Plan on gender-based violence should facilitate a consistent definition of IPV across jurisdictions and sectors that recognizes nonphysical forms of violence, as the United Kingdom and other countries have done (Koshan et al., 2020). This is especially critical in light of COVID-19 and the ways that abusive partners have used the pandemic as an additional means of coercive control, which remain outside the scope of many Canadian jurisdictions’ protective laws and interventions (Koshan et al., 2020; Koshan et al., 2021; Watson Hamilton, 2019). An amendment to the Criminal Code to include coercive control as a criminal offense was recently proposed in Canada, although whether this will be implemented and how is uncertain, nor would it necessarily lead to consistent access to different protections for IPV survivors across sectors (Koshan et al., 2021). In contrast to the United Kingdom, a national statutory definition of IPV should recognize the gendered and intersectional nature of this violence (Inter-Collegiate and Agency Domestic Violence Abuse, 2020). This is critical to ensuring that funding opportunities and interventions are responsive to the gender (and other social) inequities that produce and exacerbate violence and its health and social consequences (Daoud et al., 2016; Sev’er, 2002).

Canada's national strategies on housing and gender-based violence should be coordinated to provide the necessary legislative and funding infrastructure for key sectors to collaborate in developing, implementing, and innovating across the full continuum of intersectoral interventions. For instance, Canada's National Action Plan on gender-based violence should be developed with linkage points to the National Housing Strategy, including investment in long-term supportive housing solutions that account for the unique needs of IPV survivors (Maki, 2020; Oudshoorn et al., 2018). This must be coupled with strong monitoring and evaluation frameworks (attentive to gender-based and intersecting inequities) so that interventions can be adapted according to what works best in Canada to meet the diversity of women and gender-diverse people's needs (Dale et al., 2021). There should also be specialist training on IPV within high-yield sectors like health care, including strategies for safe identification, recording, and referral. This could be served by the curricula developed by the pan-Canadian Violence, Education, Guidance, and Action (VEGA) Project, although an evaluation of these materials is currently underway (Kimber et al., 2020). Ideally, however, this training would be further facilitated by developing coordinated systems of embedded advocates and established networks with relevant supportive services (e.g., shelter, housing, mental health, employment and income support, criminal justice), as has been shown effective internationally (Feder et al., 2011; Robbins et al., 2014). Greater coordination between sectors will allow for stronger primary prevention strategies that acknowledge the shared structural determinants of IPV, homelessness, and poor health and wellbeing outcomes. These include colonization, discrimination, trauma, social norms around gender and violence, poverty, accessible employment opportunities, and equitable service access (Bernas et al., 2019; Sev’er, 2002). In addition to investment in affordable, accessible, and safe housing for all, systemic interventions that target these social and structural determinants are key to ending gender-based homelessness and violence.

Strengthening intersectoral action to prevent gender-based homelessness and violence against women in Canada must address the disproportionate burdens faced by Indigenous women and girls due to historic and ongoing structural harms at the intersections of colonization, misogyny, and racism (Bernas et al., 2019). This includes implementing the long-called for National Action Plan on Missing and Murdered Indigenous Women and Girls (for which short-term priorities have been outlined but without timeline or budgetary commitments from the Federal Government; MMIWG National Action Plan Core Working Group, 2021) and the National Indigenous Housing Strategy, which should continue to be developed and led by Indigenous peoples. A coordinated systems approach should include IPV-housing services designed by and for Indigenous women, addressing the chronic underfunding of Indigenous services and exclusion of Indigenous understandings of homelessness and violence (Thistle, 2017), in line with the Truth and Reconciliation Commission Calls to Action (Truth and Reconciliation Commission of Canada, 2012) and the Missing and Murdered Indigenous Women and Girls Inquiry Calls for Justice (National Inquiry into Missing and Murdered Indigenous Women and Girls, 2019).

## Conclusion

The COVID-19 crisis has demonstrated the consequences of siloed and chronically underfunded social care systems worldwide. Now more than ever, policymakers must mobilize the coordination of Canada's national infrastructure to address the structural causes and outcomes of gender-based violence and homelessness. This is a critical time to draw lessons from local and international examples of coordinated intersectoral action to strengthen Canada's systems-level response to gender-based violence and homelessness—especially as the national government restarts the development of its National Action Plan for gender-based violence. Intersectoral action is essential for addressing the structural determinants of gender-based health inequities and strengthening the response to IPV during and beyond the COVID-19 pandemic.
